# Impact of investment case on equitable access to maternal and child health services in Nepal: a quasi-experimental study

**DOI:** 10.1186/s12913-021-07292-5

**Published:** 2021-12-04

**Authors:** Janak Kumar Thapa, Doris Stöckl, Raj Kumar Sangroula, Dip Narayan Thakur, Suresh Mehata, Asha Pun, Maria Delius

**Affiliations:** 1 Center for International Health, (CIHLMU),Munich, Germany, Ludwig-Maximilians-University Munich, Germany, Little Buddha College of Health Science, Kathmandu, Nepal; 2 Helmholtz Zentrum Muenchen Oberschleißheim, Munich, Germany; 3 Nepal Public Health Research and Development Center (PHRD Nepal), Kathmandu, Nepal; 4 Little Buddha College of Health Science, Kathmandu, Nepal; 5 Ministry of Health and population, Government of Nepal, Kathmandu, Nepal; 6 Health Section, UNICEF, Kathmandu, Nepal; 7 Department of Obstetrics and Gynecology, University Hospital, LMU, Munich, Germany

**Keywords:** Investment case approach, Maternal and child health, Nepal, Quasi experimental

## Abstract

**Background:**

Disparities in the use of maternal, neonatal and child health (MNCH) services remain a concern in Low- and Middle-Income countries such as Nepal. Commonly observed disparities exist in education, income, ethnic groups, administrative regions and province-level in Nepal. In order to improve equitable outcomes for MNCH and to scale-up quality services, an Investment Case (IC) approach was lunched in the Asia Pacific region. The study assessed the impact of the IC intervention package in maternal and child health outcomes in Nepal.

**Methods:**

The study used a quasi-experimental design extracting data from the Nepal Demographic Health Surveys – 2011 (pre-assessment) and 2016 (post-assessment) for 16 intervention and 24 control districts. A Difference in Difference (DiD) analysis was conducted to assess the impact of the intervention on maternal and child health outcomes. The linear regression method was used to calculate the DiD, adjusting for potential covariates. The final models were arrived by stepwise backward method including the confounding variables significant at *p* < 0.05.

**Results:**

The results of the DiD analyses showed at least four antenatal care visits (ANC) decreased in the intervention area (DiD% = − 4.8), while the delivery conducted by skilled birth attendants increased (DiD% = 6.6) compared to control area. However, the adjusted regression coefficient showed that these differences were not significant, indicating a null effect of the intervention. Regarding the child health outcomes, children with underweight (DiD% = 6.3), and wasting (DiD% = 5.4) increased, and stunting (DiD% = − 6.3) decreased in the intervention area compared to control area. The adjusted regression coefficient showed that the difference was significant only for wasting (*β* = 0.019, *p* = 0.002), indicating the prevalence of wasting increased in the intervention group compared to the control group.

**Conclusion:**

The IC approach implemented in Nepal did not show improvements in maternal and child health outcomes compared to control districts. The use of the IC approach to improve MCH in Nepal should be discussed and, if further used, the process of implementation should be strictly monitored and evaluated.

## Background

Despite the several efforts to improve the health status of mothers and children, maternal and child mortality remains a major global concern (James KS, Mishra US, Rinju, Pallikadavath S: Sequential impact of components of maternal and child health care services on the continuum of care in India, forthcoming). Goal number 3 of the Sustainable Development Goals (SDGs) aims to reduce the global maternal mortality ratio to less than 70 per 100,000 live births, neonatal mortality to less than 12 per 1000 live births and under-five mortality to 20 per 1000 live births [1, 2]. Out of total maternal deaths, 99% occur in developing countries [3, 4].

Nepal is ranked 142 out of 187 countries in terms of human development, and 34% of the people live below the poverty line [5]. The majority of the poor are women, Dalit, and disadvantaged Janjati (indigenous groups). According to the Central Bureau of Statistics [6], the most disadvantaged are households from the remote hill and mountain areas, as well as the Terai community. In Nepal, the under-5 mortality rate was reduced from 91 to 38 per 1000 during 2000–2015 period and was further reduced to 28 per 1000 in 2019 [2]. The neonatal mortality rate fell from 38 per 1000 live births in 2000 to 20 per 1000 in 2019/20 [2]. The Maternal mortality ratio (MMR) decreased from 850 per 100,000 live births in 1990 to 258 per 100,000 in 2015 and reached 239 per 100,000 live birth in 2018/19 [2]. According to the Nepal Multi Indicator Cluster Survey 2019, 77.8% of pregnant women accomplished at least four antenatal care visits (ANC), 79.3% of births were accompanied by a Skilled Birth Attendant (SBA); more than half of the newborns were exclusively breastfed (62.1%), symptoms of malnourishment were found in one third of the children: stunting (31.5%), wasting (12%) and underweight is 24.3 % [7].

To decrease the inequities in health through responsive and accessible services and an improved quality health system, the Government of Nepal (GoN) has initiated the engagement of local-level stakeholders in planning and implementing programmes, as envisioned in the Nepal Health Sector programme-Implementation Plan II [8]. The GoN is committed to bring about tangible changes in the health-sector development process and provide equitable access to quality health care for all people. The aim is to provide an equitable, high-quality health care system for all Nepalese people [9]. Maternal, Child Health and Nutrition Programs are priority programs in Nepal. The program includes different interventions such as the Safe Motherhood program, the National Immunization Program, the Newborn Care Program, and the Safe Delivery Incentive Program in all districts of Nepal [10].

**Fig. 1:**
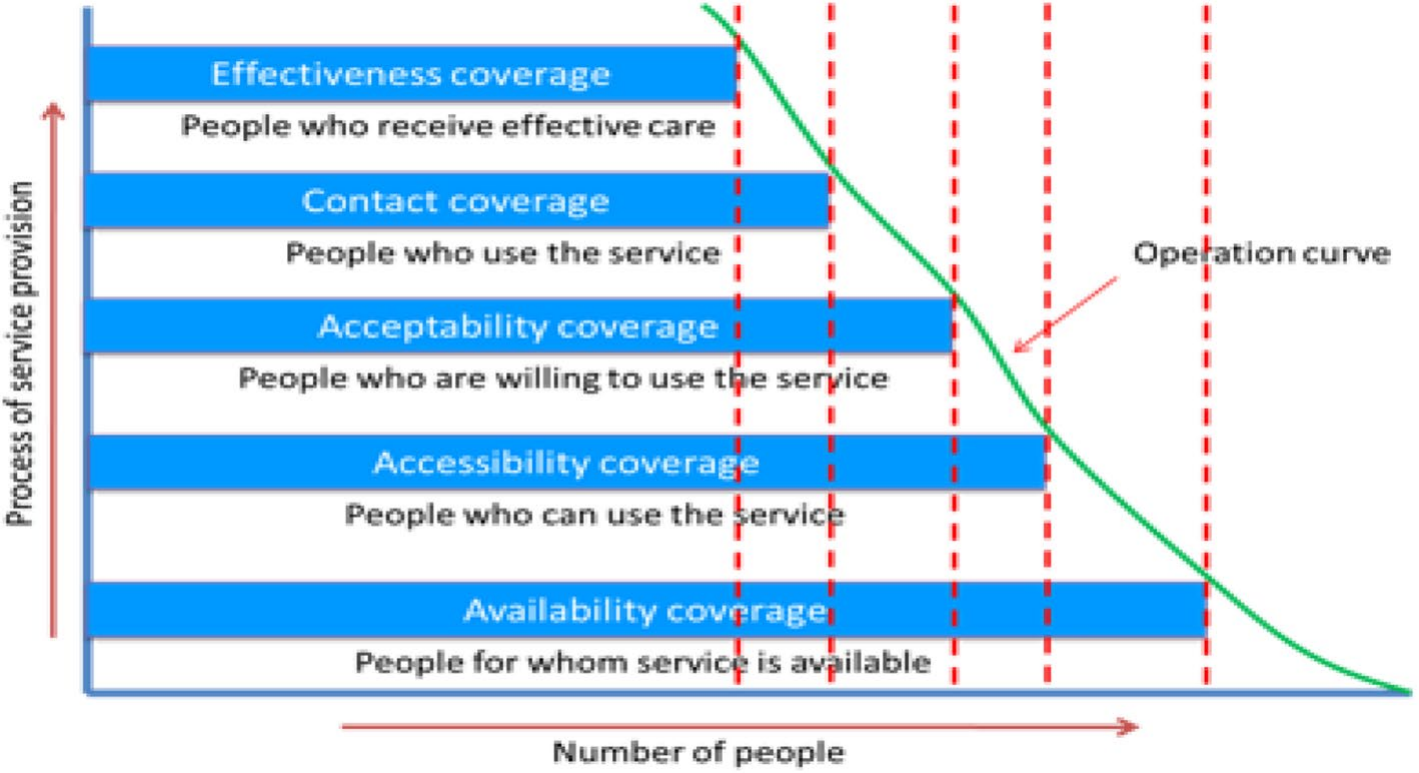
The Tanahashi Framework, illustrating the links between service delivery goals and ‘types’ of coverage [11]

The IC approach is a strategic and evidence-based problem-solving approach to support better maternal, neonatal, and child healthcare planning and budgeting. It highlights the immediate need to accelerate progress towards health-related MDGs 4 and 5 by describing health issues being faced by a country in the area of MNCH. The IC analysis is based on the ‘Tanahashi model’, bottleneck framework, which covers the idea of five different determinants to measure the capacity and intervention to produce the desired quality of service i.e., effective coverage [11]. The Tanahashi model (1978) aims to identify the gaps in the quality and effectiveness in service delivery. A gap refers to the proportion of the target population that does not receive effective coverage [11]. The five determinants of the Tanahashi model are (i) availability, (ii) accessibility, (iii) acceptability, (iv) contact, and (v) effectiveness [11]. It is designed to identify current barriers of coverage and performance and to work out the costs and impacts of potential interventions to improve performance and overall equity [11]. The implementation of the IC approach starts from advocacy with the government, selection of interventions (tracers), data mapping and collection, data validation, bottleneck analysis, and strategy development [12]. Within the IC approach the health system is examined against a range of supply, demand, and quality factors that determine the extent to which the population benefits from health services. The analysis rationale is based on the work of Tanahashi, subsequently adapted by Soucat and colleagues in the early 2000s [11, 13]. Figure 1 shows the Tanahashi Framework illustrating the links between attainment of service delivery goals and type of coverage.

The IC approach has been developed based on the five determinants described above that successively lead to a desired health intervention: The first two determinants: the availability of human resources and of essential health commodities, and the accessibility of these are supply-side determinants while the acceptability coverage, the contact coverage, and the effective coverage are demand side determinants [11]. On the supply side, the availability coverage refers to the availability of health service commodities and human resources at health facilities who provide the services related to MNCH; the accessibility coverage refers to the physical accessibility of service delivery points. On the demand side, the acceptability coverage refers to the number of people who are willing to use the accessible service; the contact coverage refers to the first contact or use of health services; and the effective coverage refers to the service performance that is appraised as satisfactory [11]. The term bottleneck is used to define particular elements that limit a whole system’s capacity to improve the health outcomes of the population [11].

### The investment case (IC) approach in Nepal

The United Nations adopted the Millennium Development Goals (MDGs) in September 2000. It was adopted in order to reduce poverty and advance other social development targets by 2015 [14]. Nepal was one of the 189 countries committed to these goals. Maternal and child health (MCH) services have been included in the basic health service package in Nepal [10]. Although Nepal had made significant progress in MCH indicators and the government of Nepal established well-developed strategies to continue the progress, the programs have not been implemented properly in every district of Nepal due to which the indicators progressed unevenly. Gaps exist regarding the commodities and trained human resources in some districts, which hamper the delivery of basic services. In order to support equitable outcomes for Maternal, Neonatal and Child Health and to scale-up the service quality, the IC approach was launched by the UNICEF and Government of Nepal in order to support the evidence-based planning, implementation, financing of the collaborative framework for health local governance and to mobilize the local resources in selected districts of Nepal from 2011 [15]. The IC approach was implemented firstly in five districts: Dadeldhura, Dhading, Kapilvastu, Jajarkot and Udayapur in 2011. It was then extended to 15 further districts for the country programme cycle of 2011–2012. In addition, the program was extended until 2016 in all the districts. The IC approach in Nepal focused on districts with a low Human Development Index (HDI) as an evidence-based planning tool to support district planning and budgeting [16]. A set of indicators had been made to monitor progress over the following year as well as in the longer term. The IC approach at the local level aims to develop a plan that is coherent with the existing local level development plans, that focuses on equitable access and responds to local bottlenecks and needs [17].

The IC program is grounded in evidence, inequity in service coverage can easily be identified through this program and targeted interventions can be designed [17]. This has helped policy and decision makers to plan and implement a health related program successfully and to reduce inequity [12]. Studies suggested that the IC approach may help in improving MCH although these studies have mostly focused on the process of implementing the IC approach [16–18]. Until now, no study evaluates the impact of the use of the IC approach in improving MCH outcomes in practice. Given this situation, this study aims to assess the impact of the IC approach on improving the access of MCH services in Nepal.

## Methods

### Study design

The study used a quasi-experimental study design to assess the impact of the intervention, the application of the IC approach on MCH services in certain districts in Nepal. The study used data from the Nepal Demographic and Health Survey (NDHS) [19, 20] for 16 intervention and 24 matched control districts. To compare MCH results pre- and post-intervention, two different data sets from NDHS surveys (NDHS 2011 and 2016) were used, with NDHS 2011 as pre- and NDHS 2016 as post-intervention observations. The intervention was implemented and carried out in the study districts by the UNICEF and Health Offices at the district level.

### Study setting and population

**Fig. 2:**
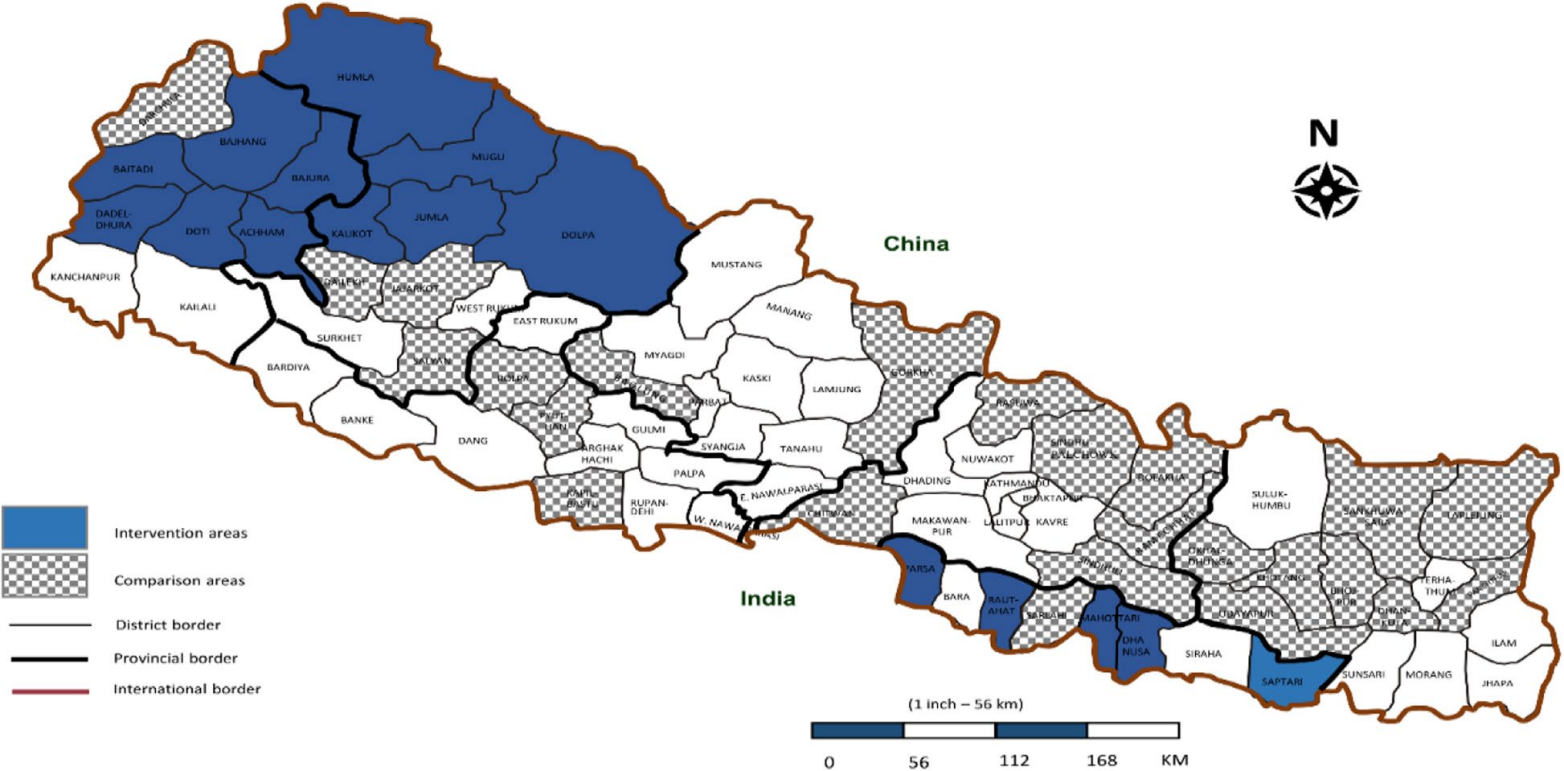
Map of Nepal showing intervention and control districts*. *The map shows 16 intervention districts and 24 control districts. The map was taken from the Wikipedia (http://en.wikipedia.org/wiki/File:Nepal_districts.png), and modified by authors using the Photoshop program

Nepal is divided into three ecological regions-mountains, hills, and plains. According to the census of 2011, 50% of the population resides in the plain area, 43% in the hills, with the remaining 7% living in mountain areas [6]. In this study, 16 districts served as intervention districts in which the Investment Case approach was implemented and 24 districts with similar HDI were taken as control districts (Fig. 2). Both intervention and control districts represented plain, hilly and mountain areas. The MCH services are one of the priority programs of the Government of Nepal with a number of programs implemented to improve MCH outcomes across all districts in Nepal. In addition to the regular government programs (in all districts), the IC approach was implemented only in the intervention districts.

### Intervention

In each of the 16 intervention districts, a district level workshop was conducted to finalize the IC approach. The intervention was carried out by the health offices at district level (Government of Nepal) in technical and financial support from the UNICEF.

The major intervention package consisted of seven components as listed below.

1. Ensuring regular supply of commodities
2. Human resources recruitment/retention and placement.
3. Capacity building of health workers: e.g., SBA for nurses, cesarean section training (advanced SBA) for doctors, essential newborn care for nurses, Community Based Integrated Maternal, Neonatal, and Childhood Illness (CB-IMNCI) and nutrition training for health workers.
4. Establishment/ strengthening of Birthing centres/Basic emergency obstetric and newborn care (BEoNC)/ Comprehensive emergency obstetric and newborn care (CEoNC) sites establishing/strengthening.
5. Regular supportive supervision and monitoring by UNICEF Nepal, district health offices.
6. Support the evidence-based planning, implementation, financing of the collaborative framework for health local governance mobilizing local resources.

During the district level workshop, detail activities for each of the components based on Tanahashi model (1978) were identified with respective action plans for the implementation.

### Data sources

The Demographic and Health Survey (DHS) is a standardized survey that collects household data on population, health, and nutrition. The DHS survey collects data from a nationally representative sample identified by multi-stage sampling [21]. The study used data from the past two surveys NDHS 2011 and NDHS 2016. Both surveys use the same methods. A detailed description of the survey design can be found in the NDHS reports [22, 23].

The sample of women represented by the NDHS reports are shown in Table 1: The number of sample households, women of reproductive age (15–49 years), and women having a child below 5 years of age in NDHS 2011 and 2016, as well as the sample of women included in this study. The sample for this study consisted of 1527 women having a child below 5 years of age in pre-assessment (679 in intervention and 848 in control) and 1343 in post-assessment (603 in intervention and 740 in control).

**Table 1:** Number of households and women of reproductive age by survey years

Parameters	NDHS 2011			NDHS 2016		
Total sample households	10,826			11,473		
*Response rate (%)*	*99.4*			*98.5*		
Total sample women aged 15–49 years	12,674			13,089		
*Response rate (%)*	*98.1*			*98.3*		
	**Intervention**	**Control**	**Total**	**Intervention**	**Control**	**Total**
Sample women for this study (women having a child below 5 years of age)	679	848	1527	603	740	1343

### Measurement and variables

**Fig. 3:**
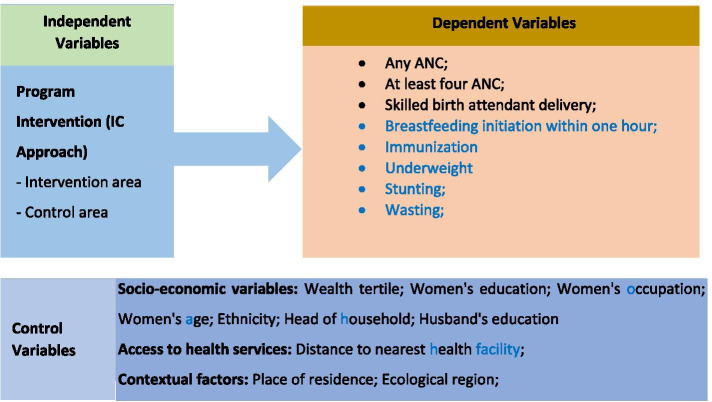
Conceptual Framework

The conceptual framework of the study is illustrated in Fig. 3: The outcome variables examined comprise: Any ANC visit, at least four ANC visits, SBA delivery, breast feeding initiation within one-hour of birth, full immunization of the child, and the child’s nutritional status which included underweight, stunting and wasting. Children whose weight-for-age Z-score, height-for-age Z-score and weight-for-height Z-score are below minus two standard deviations (− 2 SD) from the median of the reference population was classified as underweight, stunting and wasting respectively.

The control variables included in the study were the wealth tertile; women’s education; women’s occupation; place of residence; women’s age; ethnicity; distance to nearest health facility; gender of household head; ecological region; and husband’s education.

### Data analysis

Descriptive statistics (percentage and confidence intervals) were calculated for the study variables for pre- and post-assessment by intervention and control districts. The Difference in Difference (DiD) analysis was conducted to assess the impact of the intervention on MCH outcomes and to show changes in outcomes over time. The DiD analysis considers the difference between pre- and post-assessments in the study groups versus the difference between pre- and post-assessments in the control groups. This approach adjusts for time-varying factors that can influence the outcome variables [24]. The DiD analysis also addresses the bias which might occur in the sample selection since it is calculated in a regression framework to allow to control for confounders [25]. The linear regression method was used to calculate DiD, adjusting for potential covariates. We developed regression model(s) including the year of data collection (0 for 2011 and 1 for 2016), the intervention (1 for IC implemented and 0 for control districts), the interaction term between the year and the intervention, and potential covariates. The regression coefficient of the interaction between the year and the intervention provided the DiD estimating the impact of the intervention. The final models were arrived by the stepwise backward method including the confounding variables significant at *p* < 0.05. The survey data command ‘svy’ was used to adjust the complex sampling design, as it is indicated for the proper use of survey data that are not acquired by simple random sampling technique. Data were analyzed in Stata version 15.

## Results

### Socio-demographic characteristics of the study sample

Most women were from 15 to 24 years old in both intervention and control groups. The proportion of women without formal education was higher in the intervention groups, as was the proportion of women having secondary or higher-level education. Table 2 shows the socio-demographic characteristics of the study sample pre- and post-assessment by intervention and control districts.

**Table 2:** Socio-demographic characteristics of the study sample

Characteristics	2011		2016	
	Intervention (*n* = 679)	Control (*n* = 848)	Intervention (*n* = 603)	Control (*n* = 740)
	% (95% CI)	% (95% CI)	% (95% CI)	% (95% CI)
**Women’s age**				
15–24 years	51.5 (46.5–56.4)	44.7 (40.0–49.4)	51.3 (45.3–57.2)	51.4 (46.0–56.7)
25–34 years	38.1 (32.1–44.5)	43.2 (38.5–48.0)	42.8 (37.8–47.9)	41.5 (36.3–46.8)
35–49 years.	10.4 (8.02–13.4)	12.1 (9.0–16.2)	5.9 (4.0–8.5)	7.1 (4.9–10.3)
**Ethnicity**				
Advantaged	26.1 (17.8–36.6)	39.4 (32.5–46.8)	26.9 (21.6–32.9)	34.2 (26.9–42.2)
Disadvantaged	73.8 (63.4–82.1)	60.6 (53.2–67.5)	73.1 (67.1–78.4)	65.8 (57.8–73.0)
**Women’s Education**				
No Education	70.8 (61.9–78.3)	42.7 (35.7–49.9)	50.3 (42.0–58.5)	31.5 (26.6–36.7)
Primary education	12.9 (8.6–18.8)	21.2 (18.0–24.7)	19.2 (15.6–23.5)	25.5 (20.6–31.0)
Secondary or higher	16.3 (11.8–22.0)	36.1 (29.9–42.8)	30.5 (23.7–38.3)	43.0 (36.7–49.6)
**Women Occupation**				
Unemployed	38.5 (27.4–51.0)	20.3 (15.1–26.7)	51.5 (43.8–59.1)	32.8 (25.3–41.4)
Agriculture or labor work	57.2 (45.2–68.4)	71.5 (65.4–76.9)	46.0 (38.5–53.7)	58.8 (50.6–66.6)
Service or business	4.3 (2.3–7.9)	8.1 (5.8–11.4)	2.5 (1.3–4.5)	8.3 (6.2–11.1)
**Wealth tertile**				
Lowest	36.3 (28.4–45.0)	35.8 (28.8–43.3)	30.8 (25.6–36.6)	52.9 (44.5–61.0)
Middle	36.7 (31.1–42.6)	40.3 (32.8–48.4)	43.3 (38.4–48.4)	28.1 (23.7–33.1)
Highest	27.0 (20.1–35.3)	23.9 (17.8–31.2)	25.9 (21.0–31.4)	19.0 (13.0–26.7)
**Ecological region**				
Hill	19.1 (13.9–25.8)	49.9 (42.8–56.9)	13.7 (8.8–20.8)	51.3 (40.0–62.4)
Mountain	16.7 (12.4–22.3)	19.1 (14.1–25.4)	15.0 (10.1–21.7)	15.4 (8.9–25.3)
Plain	64.1 (54.1–73.0)	31.0 (24.9–37.8)	71.3 (65.7–76.3)	33.3 (23.9–44.2)
**Place of residence**				
Urban	28.1 (17.0–42.7)	49.0 (35.0–63.0)	42.2 (29.4–56.0)	51.4 (39.6–63.0)
Rural	71.9 (57.3–82.9)	51.0 (36.9–64.9)	57.8 (44.0–70.5)	48.6 (37.0–60.4)
**Husband education**				
No education	35.7 (28.1–44.1)	23.8 (17.6–31.2)	19.8 (14.8–25.8)	14.8 (11.6–18.7)
Primary education	21.9 (17.7–27.0)	23.9 (19.7–28.8)	24.4 (19.5–30.0)	26.5 (21.7–32.0)
Secondary or higher	42.3 (35.9–48.9)	52.3 (46.8–57.7)	55.8 (48.5–62.9)	58.7 (52.9–64.3)
**Gender of household head**				
Male	78.8 (68.8–86.3)	74.8 (69.9–79.1)	75.4 (69.3–80.6)	70.7 (66.4–74.7)
Female	21.1 (13.7–31.2)	25.2 (20.9–30.1)	24.6 (19.4–30.7)	29.3 (25.3–33.6)

### Changes in the outcome variables over time

MCH outcome variables pre- and post-assessment by intervention and control districts are shown in Table 3, as well as the results of the DiD analysis (adjusted regression coefficients) estimating the impact of the intervention. After the intervention period, all three maternal health outcomes (any ANC visit, at least 4 ANC visits and SBA birth) improved in both intervention and control groups over time. Regarding the child health outcomes, the changes over time were variable. The initiation of breastfeeding within 1 h of birth improved in both groups, while the proportion of fully immunized children decreased in both groups over time. There were variations in the changes in the child nutrition outcomes between intervention and control groups. The prevalence of stunting decreased in both groups, while the prevalence of underweight and wasting increased in the intervention area and decreased in control area.

**Table 3:** Difference in difference analysis of intervention on MCH outcomes

Outcome variables	Intervention area (%)			Control area (%)			Effect of the intervention (DiD)		
	Pre	Post	Difference	Pre	Post	Difference	Change over time (%) between intervention and control	DiD (*β*)	*p*-value
Any ANC	82.4	95.3	12.9	81.6	93.6	12.0	0.9	−0.004^a^	0.641
At least 4 ANC	40.1	52.5	12.4	43.9	61.1	17.2	−4.8	−0.022^b^	0.062
Skilled birth delivery	30.1	51.0	20.9	28.8	43.1	14.3	6.6	0.001^c^	0.939
Breast feeding within one hour of birth	38.8	54.2	15.4	43.5	59.1	15.6	−0.2	−0.005^d^	0.726
Full immunization	55.8	51.3	−4.5	63.6	57.4	−6.2	1.7	−0.020^e^	0.129
Stunting	46.9	38.2	−8.7	45.4	43.0	−2.4	−6.3	−0.006^f^	0.565
Underweight	35.9	38.2	2.3	33.8	29.8	−4.0	6.3	0.015^g^	0.134
Wasting	10.9	14.8	3.9	12.4	10.9	−1.5	5.4	0.019^h^	0.002

*β*: Adjusted regression coefficient denoting the difference in difference between intervention and control groups.

^a^ Adjusted for wealth index, place of residence, women age, distance from health facility, and husband education

^b^ Adjusted for wealth index, women education, women occupation, place of residence, ethnicity, gender (head of household), ecological region, and husband education

^c^ Adjusted for wealth index, women education, place of residence, women age, distance from health facility and ecological region

^d^ Adjusted for women education, place of residence, ecological region and husband education

^e^ Adjusted for distance from health facility and husband education

^f^ Adjusted for wealth index, women education, child age

^g^ Adjusted for wealth index, women education, women age and ecological region

^h^ Adjusted for wealth index, women education, women age and ecological region

### Impact of the IC approach on the outcomes

The results of the DiD analyses showed that the changes over time having at least 4 ANC visits were in favour of the control area, which means, that the positive change that occurred in both areas pre- and post-intervention was less in the intervention area (DiD% = − 4.8). The variables “delivery conducted by SBA” and “any ANC visit” increased (DiD% = 6.6) over time in all districts, while the pre- and post-intervention change was higher in the intervention districts compared to the control districts. However, the adjusted regression coefficient showed that all these differences were not statistically significant, indicating a null effect of the intervention. Regarding the child health outcomes results varied greatly. In all districts less children were fully immunized at the second time point with intervention districts having a slightly better proportion of immunized children (DiD% = 1.7) without any statistical difference between groups. The variable “stunting” decreased in both groups over time, while the time effect was greater in the intervention group (DiD% = − 6.3), this difference stayed statistically insignificant. The variables “underweight” (DiD% = 6.3), and “wasting” (DiD% = 5.4) increased in the intervention area while they decreased in the control area over time. The adjusted regression coefficient showed that the difference was statistically significant only for the variable “wasting” (*β* = 0.019, *p* = 0.002), indicating the prevalence of wasting in children increased significantly in the intervention group compared to the control group (Table 3).

## Discussion

This study utilized data of the NDHS 2011 and 2016 to evaluate the impact of the IC approach in districts where the approach was implemented. The results showed that maternal health outcomes such as utilizing ANC services, and delivery attended by SBA improved in both intervention and control districts over time. Among the child health related indicators, improvements over time were observed in both areas with less children stunting. The prevalence of underweight and wasting, however, increased in the intervention area, the proportion of children not being fully immunized increased in both, the control and the intervention area. The DiD analysis, in general, showed a null effect suggesting that there was no statistically significant difference in MCH indicators over time in districts where the IC approach was implemented in comparison to the control districts.

Though the control districts did not receive the DIC planning support as well as the support for the implementation of the DIC plans, they still had good or even better MCH indicators. This could be because both intervention and control groups had strong MCH interventions implemented by the National Government through the district health system, since MCH services have high priority for the GoN [10]. This might be the reason for the comparable good results in the control districts as they were also exposed to these priority programs since they are prevalent all over the county. Regarding MCH outcomes this study found no additional positive impact of the IC approach. Given this result, implementing the IC approach with the aim to reach improvement in MCH in Nepal seems not to work successfully.

The result obtained in this study contradicts with the results of a qualitative study conducted among the stakeholders and beneficiaries concerning the perception of the IC approach for equitable access to maternal neonatal and child health services in Nepal by the same study group [26]. There it could be seen that the players felt that the IC approach had supported them to prepare an evidence-based plan in an interactive way involving relevant stakeholders.

The IC approach is a complex in its nature requiring different interventions related to various maternal, neonatal, and child health-related indicators. The evaluation of the IC approach in Asian-Pacific countries had also shown that the complexity of the approach was too high. The district managers perceived the effective implementation of the IC tool to be beyond the staff’s capacity [12].

The IC approach in Nepal focused primarily on budgeting and capacity building at the district level. During the workshops conducted in the districts action plans were developed specifying the responsibilities of the different stakeholders. However, the performance of the action plans was not monitored on a regular basis. Resource limitations also played a major role in the quality of the intervention as the hard to reach areas had limited resources (especially human resources) to carry out the activities [12]. Some of the outcomes showed to be even worse in both areas at the second time point. This means all existing measures to improve MCH should be reviewed.

One possible explanation that the IC approach did not work in the field of MCH could lie in the complexity of the approach itself. The approach is a theoretical framework, which can help to find limitations of a health system in theory, looking for the “bottlenecks”. In practice, the approach does not seem to work as easily, as the implementation may be a too complex process especially in remote areas. On the other hand, this theoretical framework could be not flexible enough to react to sudden changes in the environment which could change the situation in a way that the before identified bottleneck is no longer an important gap and the main problems are to be sought somewhere else.

The study has a number of strengths. The study used data from nationally representative NDHS surveys with a large sample size, which itself had a rigorous and scientific design. The study included a wide range of maternal and child health indicators as outcome variables and used appropriate an statistical analysis including appropriate weighing. Another strength is the authors being independent of the implementation of the IC approach, so there is no examiner’s bias to be expected. The major limitations of the study are related to the nature of the study design and no detail information on monitoring and implementation aspects of intervention (i.e. IC approach). Since the study team did not control the implementation of the IC approach, this study pictures a “real life” situation and not a “optimal lab” situation. From the theoretical point of view, it could be seen as a limitation that the implementation of the intervention (e.g., dose and intensity) was not controlled by the study team. As there were limited previous studies on the impact of IC approach on MCH outcomes, we could not compare the results of this study with other similar studies. Further studies should be conducted in other countries and settings to assess the impact of IC approach on MCH outcomes. Future studies could overcome the limitations of this study by implementing strong study design (preferably a Randomized Controlled Trial) to assess the impact of IC approach intervention on health outcomes.

## Conclusion

The IC intervention was implemented based on Tanahashi model (1978) to support equitable outcomes for maternal, neonatal and child health and to scale-up quality services aiming to identify the gaps in the quality and effectiveness in service delivery. The IC approach was launched by developmental partners to enhance the evidence-based planning, implementation, financing of the collaborative framework for health local governance and to mobilize the local resources in selected districts of Nepal. The findings showed that the IC approach did not show improvements in MCH outcomes compared to the control districts. This leads to the conclusion that there may be better approaches to improve MCH in Nepal and that financial resources can be better used in other measures. In the hard-to-reach areas other less complex plans than the IC approach could lead to better MCH results. If the IC approach may be used more successfully, it may need some modification within the process, requires good monitoring of the implementation of the plans and a regular supportive and feedback mechanism.

## Data Availability

The datasets used and/or analyzed during the current study available from the corresponding author on reasonable request.

## Abbreviations

ANC: Ante natal checkup
CI: Confidence interval
DHS: Demographic health survey
DiD: Difference in difference
GoN: Government of Nepal
HDI: Human development index
HF: Health facility
IC: Investments case
LMU: Ludwig-Maximilians university
MDG: Millennium development goals
MCH: Maternal and child health
NDHS: Nepal demographic and health survey (NDHS)
NHSP-IP II: Nepal health sector programme-implementation plan II
SBA: Skill birth attendance
UNICEF: United Nation children’s emergency fund
